# *Eucommia ulmoides* Ameliorates Glucotoxicity by Suppressing Advanced Glycation End-Products in Diabetic Mice Kidney

**DOI:** 10.3390/nu10030265

**Published:** 2018-02-26

**Authors:** Moon Ho Do, Jinyoung Hur, Jiwon Choi, Mina Kim, Min Jung Kim, Yoonsook Kim, Sang Keun Ha

**Affiliations:** 1 Korea Food Research Institute, 245, Nongsaengmyeong-ro, Iseo-myeon, Wanju_Gun, Jeollabuk-do 55365, Korea; Do.Moon-ho@kfri.re.kr (M.H.D.); jyhur@kfri.re.kr (J.H.); Choi.Ji-won@kfri.re.kr (J.C.); Kim.Mi-na@kfri.re.kr (M.K.); kmj@kfri.re.kr (M.J.K.); 2 Divison of Food Biotechnology, University of Science and Technology, Daejeon 305-350, Korea

**Keywords:** glucotoxicity, advanced glycation end-product, nuclear factor erythroid 2-related factor 2, glyoxalase

## Abstract

*Eucommia ulmoides* Oliv. (EU), also known as Du-Zhong, is a medicinal herb commonly used in Asia to treat hypertension and diabetes. Despite evidence of the protective effects of EU against diabetes, its precise effects and mechanisms of action against advanced glycation end-products (AGEs) are unclear. In this study, we evaluated the effects of EU on AGEs-induced renal disease and explored the possible underlying mechanisms using streptozotocin (STZ)-induced diabetic mice. STZ-induced diabetic mice received EU extract (200 mg/kg) orally for 6 weeks. EU treatment did not change blood glucose and glycated hemoglobin (HbA1c) levels in diabetic mice. However, the EU-treated group showed a significant increase in the protein expression and activity of glyoxalase 1 (Glo1), which detoxifies the AGE precursor, methylglyoxal (MGO). EU significantly upregulated nuclear factor erythroid 2-related factor 2 (Nrf2) expression but downregulated that of receptor for AGE (RAGE). Furthermore, histological and immunohistochemical analyses of kidney tissue showed that EU reduced periodic acid–Schiff (PAS)-positive staining, AGEs, and MGO accumulation in diabetic mice. Based on these findings, we concluded that EU ameliorated the renal damage in diabetic mice by inhibiting AGEs formation and RAGE expression and reducing oxidative stress, through the Glo1 and Nrf2 pathways.

## 1. Introduction

Diabetes mellitus is one of the most complicated diseases, and its etiology is not yet thoroughly understood. Diabetes mellitus can cause secondary complications such as heart disease, stroke, retinopathy, nephropathy, and neuropathy. Although various factors can complicate diabetes, the increase in advanced glycation end-product (AGE) formation in chronic hyperglycemic condition is regarded as the main contributor [1]. AGEs can be formed endogenously via a non-enzymatic glycation reaction between reducing sugars and the free amino groups of proteins or lipids [2]. This non-enzymatic reaction of proteins and lipids leads to the formation of AGEs by molecular rearrangements. AGEs induce oxidative stress, inflammatory reactions, and thrombosis by interacting with their receptors (RAGEs) [3]. Therefore, they are involved in vascular aging, cataract generation, retinopathy, atherosclerosis, and nephropathy [1]. Moreover, AGEs accumulate in the glomeruli where they increase the expression of type-IV collagen and laminin in the extracellular matrices [4] and induce irreversible cross-linked protein formation [5].

Methylglyoxal (MGO) is one of the most significant reactive carbonyl species and the major precursor in the formation of AGEs [6]. MGO-induced dicarbonyl stress may induce aging, retinopathy, atherosclerosis, and renal dysfunction [7]. Numerous studies have shown that long-term hyperglycemia increase MGO accumulation in the kidney of diabetic animal models [8].

The glyoxalase (Glo) system is known to play a critical role in the detoxification of AGEs. The Glo system comprises two enzymes, glyoxalase 1 (Glo1) and Glo2. The highly expressed and activated Glo1 detoxifies MGO and glyoxal (GO) and directly inhibits AGEs formation [9,10]. Numerous research studies have reported that overexpression of Glo1 reduces hyperglycemia-induced AGE levels and oxidative stress in diabetic rodents [11]. Nuclear factor erythroid 2-related factor 2 (Nrf2) is an essential component of the antioxidant responsive element, which mediates the induction of antioxidant enzymes and increases the expression of Glo1 [12]. Downregulation of Glo1 in diabetes is likely due to the inhibition of Nrf2 expression [13].

Inhibition of AGE accumulation or its breakdown has been shown to be a useful strategy for inhibiting or delaying diabetic nephropathy [14]. Currently, no significantly effective drugs are available to inhibit AGEs formation or breakdown. Although aminoguanidine and the thiazolium-derived compound ALT-711 (alagebrium) showed promising effects, safety and efficacy concerns have led to the discontinuation of the clinical development of these candidate drugs [15].

*Eucommia ulmoides* Oliver (EU), also known as Du-Zhong, is a commonly used medicinal herb in Asia for the treatment of hypertension and diabetes. The antioxidant, anti-hypertensive, and anti-hyperglycemic effects of EU have been demonstrated [16,17,18]. Moreover, EU has been used as a functional food that reinforces the muscles, improves liver and kidney tone, and increases longevity [16]. EU contains various phytochemicals including lignin, phenolics, terpenoids, and flavonoids [19]. These components are known to be effective in preventing diabetes and inhibiting AGEs. Moreover, Sugawa et al. [20] reported that EU leaf extract inhibited the formation of N^ε^-(carboxymethyl)lysine and N^ω^-(carboxymethyl)arginine. Based on these observations, we hypothesized that the bark of EU has protective effects on AGEs-induced renal glucotoxicity. Therefore, we investigated the inhibitory effects of EU on AGEs formation and AGEs cross-links and its protective effects against AGEs-induced renal damage in vitro and in vivo.

## 2. Materials and Methods

### 2.1. Materials

Glucose, fructose, bovine serum albumin (BSA), sodium azide, phosphatase inhibitor cocktail, and streptozotocin (STZ) were purchased from Sigma-Aldrich (St. Louis, MO, USA). Antibodies against RAGE, Nrf2, lamin B, β-actin, Glo1, and secondary antibodies were purchased from Cell Signaling Technology (Danvers, Ma, USA). The AGEs and MGO antibodies were obtained from Abcam (Cambridge, MA, USA). Nuclear/cytosol fraction and Glo1 activity assay kits were obtained from Biovision (Milpitas, CA, USA).

### 2.2. Sample Preparation

The bark of EU was obtained from Gyeongdong Market in Seoul, Korea and a voucher specimen (H-378) was deposited at the Korea Food Research Institute. The EU bark was extracted with 70% ethanol at room temperature overnight, filtered, evaporated, and then concentrated, followed by lyophilization using a freeze-dryer. The final product was stored at −20 °C until used and the yield of the EU extract was 11.5%.

### 2.3. Ultraperformance Liquid Chromatography (UPLC)-Tandem Mass Spectrometry (MS/MS) Analyses

The ultraperformance liquid chromatography (UPLC)-tandem mass spectrometry analyses were performed using an Acquity UPLC system (Waters, Milford, MA, USA) coupled with a Waters Xevo TQ triple-quadrupole mass spectrometer equipped with an electrospray ionization (ESI) source. MassLynx 4.1 (Waters) was used for data processing. The mobile phase consisted of a 0.1% formic acid aqueous solution (solvent A) and 0.1% formic acid in acetonitrile (solvent B). The gradient elution was performed using an Acquity UPLC BEH C18 column (2.1 mm × 100 mm, 1.7 µm) on the following schedule: 0–3 min, 95–50% solvent A; 3–4.5 min, 50–20% solvent A; 4.5–5 min, 20–5% solvent A; 5–5.5 min, 5% solvent A; and 5.5–6.5 min, 5–95% solvent A. The column temperature was maintained at 25 °C at a flow rate of 0.3 mL/min. The auto-sampler was conditioned at 4 °C, and the injection volume was 3 µL. The LC-MS/MS system was operated in the negative ESI mode, and multiple reaction monitoring (MRM) mode was used to scan the geniposidic acid. The MRM transitions were monitored at *m*/*z* 373.1 → *m*/*z* 211.1 for geniposidic acid. The voltage of the capillary, cone and collision energy was set at 3.2 kV, 34 V, and 22 V, respectively. The gas flow for desolvation and the cone was 800 and 50 L/h, respectively. The source and desolvation gas temperatures were 150 °C and 400 °C, respectively.

### 2.4. Inhibitory Effects of EU on AGE Formation

BSA (10 mg/mL) was incubated with glucose and fructose (25 mM) in phosphate-buffered saline (PBS, pH 7.4) in the presence or absence of EU (10, 50, and 100 μg/mL). Sodium azide (0.02%) was added to the reaction mixture, which was incubated at 37 °C for 14 days. The formation of AGEs was determined using fluorescence analysis at an excitation/emission wavelength of 350/450 nm using a microplate reader (Molecular Devices, Sunnyvale, CA, USA).

### 2.5. AGE Cross-Link Inhibitory Effect of EU

The inhibition assay was performed according to the method of Lee et al. [21] with slight modifications. To confirm the inhibition of AGEs-BSA-induced cross-links to collagen by EU, 1 mg/mL of AGEs-BSA was incubated with or without AG or EU on collagen-coated 96-well plates at 37 °C for 24 h. The inhibitory ability of EU was detected using tetramethylbenzidine (TMB) as the substrate. The inhibition of AGEs-induced cross-links was expressed as the percentage decrease in optical density.

### 2.6. Animals

Six-week-old male C57BL/6 mice were purchased from Joongang Laboratory Animal Inc., (Seoul, Korea). The mice were housed at 23 °C in a 65% humidity-controlled animal room on a 12-h light/dark cycle. The animals were provided food and water *ad libitum*. All animal experiments were performed in accordance with the guidelines for Animal Care and Use Committee of the Korea Food Research Institute, approval number KFRI-M-17006.

After 1 week acclimatization, the mice were injected intraperitoneally with STZ at 50 mg/kg daily for 5 days while the control mice received the vehicle alone. One week after the last injection of STZ, mice with blood glucose levels > 200 mg/dL were considered diabetic. The mice were subsequently randomly divided into the following four treatment groups (*n* = 8): (1) normal control mice (Normal); (2) diabetes control (Control); (3) aminoguanidine (AG, 200 mg/kg, oral administration); and (4) EU extract (EU, 200 mg/kg, oral administration). The EU extract and AG were dissolved in distilled water and were orally administered by gavage while the Normal group was administered a similar amount of the vehicle for 6 weeks.

### 2.7. Blood Measurements

We obtained blood from the tail vein. Fasting blood glucose and HbA1c levels were determined using a glucometer (Accu-chek, Roche Diagnostics, Basel, Switzerland) and an HbA1c analyzer (Infopia Inc., Anyang, Korea), respectively according to the manufacturer’s instructions.

### 2.8. Measurement of Total AGEs

Serum and kidney total AGEs levels were measured using enzyme-linked immunosorbent assay (ELISA) kits (Cell Biolabs, Inc., Beverly, MA, USA) according to the manufacturer’s instructions and the results were normalized to total protein concentrations. Briefly, 50 μL of samples or the AGEs-BSA standard was added to the wells of the AGE conjugate-coated plate and 50 μL anti-AGE antibody was added, followed by incubation for 1 h at room temperature. Then, 50 μL of the secondary antibody-horseradish peroxidase (HRP) conjugate was added to the plate and incubated at room temperature for 1 h. After washing three times, 50 μL substrate solution was added to the plate, which was incubated for 5 min protected from light and then 100 μL stop solution was added. The total AGE amount was measured using a microplate reader at 450 nm.

### 2.9. Western Blotting Analysis

Total proteins from the kidney tissue were extracted using PRO-PREP^TM^ (iNtRON Biotechnology, Seongnam, Korea) containing a phosphatase inhibitor. Equal amounts of protein were loaded onto a 10% sodium dodecyl sulfate-polyacrylamide gel electrophoresis (SDS-PAGE) gel and transferred onto membranes, which were blocked with 5% skim milk for 1 h at room temperature and incubated with the primary antibodies overnight at 4 °C. Then, the membranes were incubated with peroxidase-labeled secondary antibodies for 1 h at room temperature, and the immunoreactive proteins were visualized using enhanced chemiluminescence (ECL) reagents using a ChemiDoc XRS+ imaging system (Bio-Rad, CA, USA).

### 2.10. Glo1 Activity Assay

The Glo1 activity was measured using a Glo1 activity kit (QuantiChrom^TM^ Glo1 assay kit, BioAssay Systems, Hayward, CA, USA) according to the instructions of the manufacturer. Briefly, 40 μL samples were mixed with 160 μL working reagent, incubated for 20 min at room temperature, and then 70 μL 4 M perchloric acid was added to each sample tube, followed by vortexing and then the mixture was chilled for 15 min on ice. Then, the samples were centrifuged at 12,000 rpm for 10 min, 200 μL of each sample supernatant was added to a 96 well plate, and the Glo1 activity was read by measuring the optical density at 240 nm using the microplate reader.

### 2.11. Histology and Immunohistochemistry

The kidney tissue samples were fixed in 4% formaldehyde for 24 h, embedded in paraffin, and then 4-μm-thick sections were cut and stained with periodic acid–Schiff (PAS) for mesangial expansion and glycogen accumulation measurement.

To monitor the AGE and MGO accumulation, 3-μm-thick sections of the kidney tissues were used. Antigen retrieval was performed using 20 μg/mL proteinase in PBS for 20 min at 37 °C. The sections were then incubated with 3% hydrogen peroxide (H_2_O_2_) in PBS for 15 min to quench the endogenous peroxidase activity. The sections were incubated with primary antibodies against AGEs (1:500) or MGO (1:100) for 12 h at 4 °C. After washing in PBS, the sections were incubated with secondary antibodies (1:200) for 20 min at room temperature, rinsed twice with PBS, and then incubated in Vectastain ABC reagent (Vector Laboratory, Piscataway, NJ, USA) for 30 min. Immunoreactions were visualized using 3,3′-diaminobenzidine (Vector Laboratory, Piscataway, NJ, USA). The sections were counter-stained with hematoxylin for 3 min, and all sections were scanned using the Panoramic 250 Flash III slide scanner (3DHistech, Ltd., Budapest, Hungary) and analyzed using the CaseViewer software (3DHistech, Ltd., Budapest, Hungary). Renal morphometric changes were observed in 10 randomly selected glomeruli per mouse.

### 2.12. Statistical Analysis

The statistical analyses results are presented as the means ± standard error of the mean (SEM) and were analyzed using a one-way analysis of variance (ANOVA) followed by Tukey’s test. A *p*-value < 0.05 was considered statistically significant. All the data were analyzed using GraphPad Prism 5 (GraphPad Software, Inc., San Diego, CA, USA).

## 3. Results

### 3.1. Geniposidic Acid Concentration in EU

**Figure 1:**
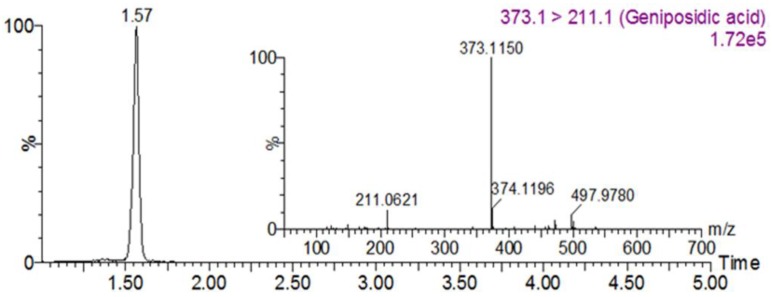
Chromatogram of *Eucommia ulmoides* Oliv. (EU) extracts.

He et al. [19] reported the isolation and identification of 24 iridoids from EU [19]. Among these compounds, geniposidic acid has been confirmed to possess various pharmacological effects. Based on a UPLC-MS/MS analysis, the concentration of geniposidic acid in EU was 77.0 ± 0.26 mg/g (Figure 1).

### 3.2. Inhibitory Effect of EU on AGE Formation and Cross-Linking

**Figure 2:**
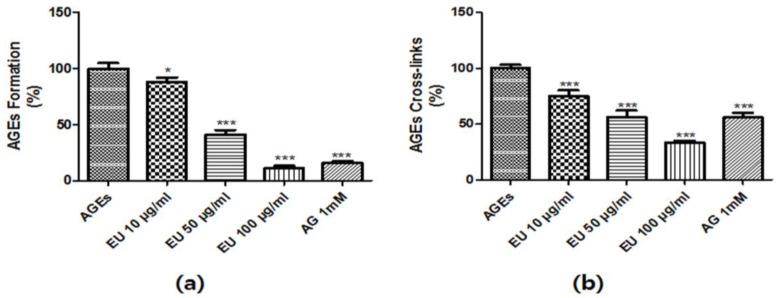
The inhibitory effect of *Eucommia ulmoides* Oliv. (EU) on advanced glycation end-products (AGEs) formation and AGEs-induced cross-link to collagen. (**a**) The effects of EU on in vitro AGEs formation was examined using an AGEs formation assay. Bovine serum albumin (BSA, 5 mg/mL) was incubated with 25 mM of glucose and fructose in the presence or absence of EU in phosphate-buffered saline (PBS) for 14 days; (**b**) AGEs cross-linking inhibition by EU was evaluated by determining the optical density of AGEs-collagen cross-linking using tetramethylbenzidine (TMB) as the substrate. The results of each experiment are presented as the mean ± SD (%) of three independent experiments for three repeat experiment on each sample (* *p* < 0.05 and *** *p* < 0.001 vs. AGEs).

To investigate the inhibitory effect of EU on AGEs formation, fluorescence excitation and emission spectra were monitored at 350 nm and 450 nm, respectively. As shown in Figure 2, AGEs formation was induced by BSA-glucose and fructose incubation. However, EU treatment (10, 50 and 100 μg/mL) significantly inhibited the formation of AGEs in a dose-dependent manner (Figure 2A).

We investigated the inhibitory effects of EU on the AGE-collagen cross-linking using ELISA. AGEs-BSA treatment significantly increased AGEs-BSA-induced cross-links to collagen. However, AGEs-collagen cross-linking was markedly reduced by EU in a dose-dependent manner (Figure 2B). Taken together, these results indicate that EU appeared to not only inhibit AGEs formation but also contributed to the inhibition of AGEs-collagen cross-linking.

### 3.3. Effects of EU on Diabetic Parameters

**Figure 3:**
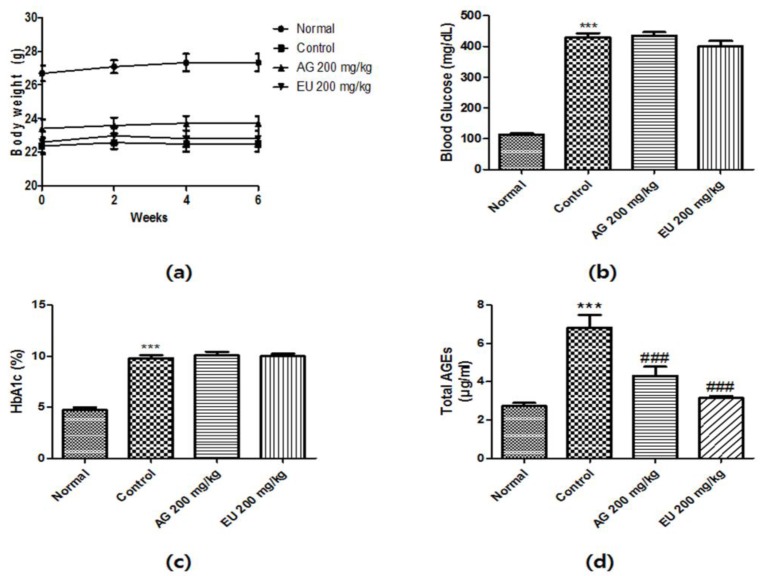
Diabetic parameters of streptozotocin (STZ)-induced diabetic mice. (**a**) Time course of body weight changes of mice; (**b**) Fasting blood glucose levels of normal and STZ-induced diabetic mice following a 6-week treatment and after 12 h of fasting; (**c**) Glycated hemoglobin (HbA1c) levels of normal and STZ-induced diabetic mice after a 6-week treatment; (**d**) Serum advanced glycation end-product (AGE) levels of normal and STZ-induced diabetic mice after a 6-week treatment and 12 h fast. Value are means ± standard error of the mean (SEM, *** *p* < 0.001 vs. normal and ### *p* < 0.001 vs. control).

As shown in Figure 3, the body weight of the STZ-induced diabetic mice significantly decreased compared with that of the normal mice. The EU-treated mice did not show improvements in the diabetes-induced decrease in body weight. The levels of fasting blood glucose and HbA1c were assessed on day 42 after overnight fasting. A significant increase in fasting blood glucose and HbA1c levels was observed in diabetic mice compared to levels in the normal mice. However, EU treatment did not affect blood glucose and HbA1c levels (Figure 3b,c). As shown in Figure 3d, serum AGEs levels significantly increased in the STZ-induced diabetic mice, but EU treatment effectively reduced this pattern.

### 3.4. Effects of EU on Glo1 Expression and Activity

**Figure 4:**
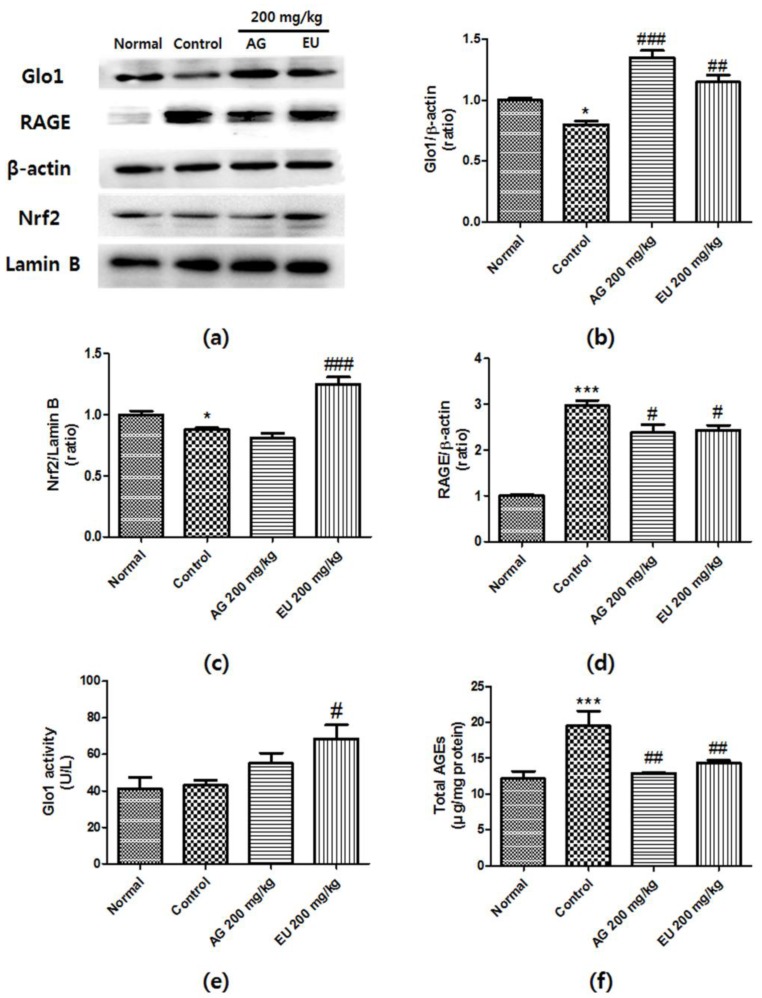
The effects of *Eucommia ulmoides* Oliv. (EU) on the expression of glyoxalase 1 (Glo1), receptors of advanced glycation end-product (RAGE), and nuclear factor erythroid 2-related factor 2 (Nrf2), and Glo1 activity. (**a**) Representative western blots of Glo1 are shown. β-Actin and lamin-B were used as internal controls. Relative band intensities of (**b**) Glo1; (**c**) RAGE; and (**d**) and nuclear Nrf2. The experiments were repeated three times, and one representative blot is shown. (**e**) GLO-1 activity of STZ-induced diabetic mouse kidney tissue was determined by measuring the formation of *S*-d-lactoylglutathione; (**f**) Total AGE levels in mouse kidneys were measured using enzyme-linked immunosorbent assay (ELISA) of AGEs-protein adducts. Values are means ± standard deviation (SD) of three independent experiments; (* *p* < 0.05 and *** *p* < 0.001 vs. normal; # *p* < 0.05, ## *p* < 0.01, and ### *p* < 0.001 vs. control).

Glo1 is well known as a key enzyme that regulates AGEs by detoxifying MGO. To examine whether EU activates the Glo pathway, we performed western blot analysis and a Glo1 activity assay. As shown in Figure 4b, Glo1 expression was reduced in the diabetic mouse kidney tissues; however, this effect was attenuated by EU treatment. Moreover, supplementation with EU significantly increased Glo1 activity in the diabetic kidney tissue (Figure 4c). These results indicate that EU treatment increased Glo1 expression and activity, thereby reducing AGE accumulation in the kidney.

### 3.5. Effects of EU on Nrf2 and RAGE Expression

The activation of the Nrf2 system increases Glo1 expression. Using western blot analysis, we investigated whether EU affects the activation of Nrf2 in the diabetic kidney. Figure 4c shows that the diabetic mouse group exhibited significantly reduced levels of Nrf2 in the nuclei. Notably, this effect was attenuated by EU treatment. As shown in Figure 4d, RAGE expression significantly increased in the diabetic mouse group compared with that in the normal group. However, treatment with EU downregulated RAGE protein expression. Moreover, the total AGE amount in the kidney also significantly increased, but EU treatment reversed this effect. These findings suggest that EU treatment ameliorated the diabetes-induced kidney damage by reducing RAGE expression and AGE accumulation through activation of the Nrf2-Glo1 interaction.

### 3.6. Histology and Immunohistochemistry

**Figure 5:**
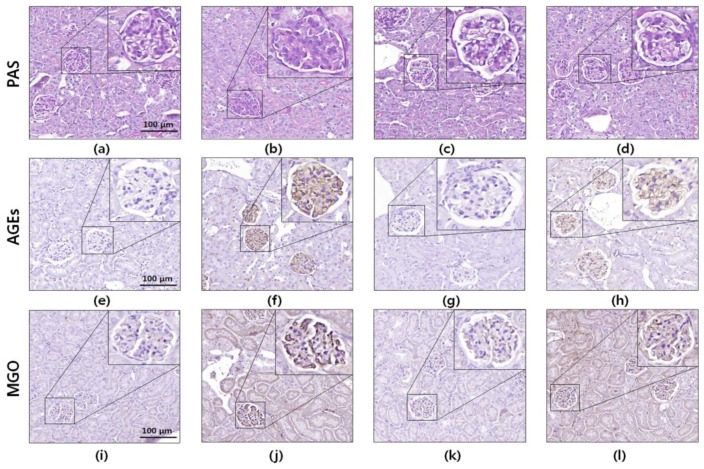
Periodic acid-Schiff (PAS) and immunohistochemical (IHC) staining of kidney tissues of STZ-induced diabetic mice. Representative photomicrographs of (**a**–**d**) PAS-stained kidney tissue; (**e**–**h**) immunohistochemical analysis of advanced glycation end-products (AGEs); and (**i**–**l**) methylglyoxal (MGO) in kidney sections from mice. The images shown are representative (**a**,**e**,**i**) normal; (**b**,**f**,**j**) STZ-induced diabetic; (**c**,**g**,**k**) aminoguanidine-treated diabetic; and (**d**,**h**,**l**) *Eucommia ulmoides* Oliv. (EU)-treated diabetic mouse kidney tissues. The histological sections stained with PAS and analyzed using IHC are presented at 400× magnification.

The diabetic kidney tissues showed diverse histological features such as glomerular mesangial expansion, podocyte loss, and glomerular basement membrane thickening. To investigate the potential protective effects of EU against renal damage, PAS staining and immunohistochemistry were performed using kidney tissue sections. Histochemical observations did not show glomerular mesangial expansion in the kidney tissues of the diabetic mice. However, the PAS-positive staining (glycated protein) markedly increased in the diabetic mouse kidney tissue, and this pattern was significantly attenuated by EU treatment (Figure 5).

Immunohistochemical analysis of the kidney tissue sections revealed that AGE expression in the glomeruli significantly increased in the diabetic mice compared to that in the normal group. Increased renal AGE accumulation was markedly reduced by EU treatment. Moreover, the glomeruli of the diabetic mice showed elevated accumulation of MGO, a precursor of AGE, but EU treatment effectively reduced the MGO overexpression. These results indicate that the diabetes-induced AGEs formation and accumulation in the kidney were dramatically controlled by EU treatment.

## 4. Discussion

Elevated AGE levels play an important role in the progression of diabetic nephropathy [22]. In the glomeruli and tubulointerstitium in experimental and diabetic nephropathy, AGE accumulation can be characterized based on the severity of renal disease [4]. Additionally, AGEs induce reactive oxygen species (ROS) production in kidney cells that might also be deleterious to kidney health [23]. Hence, reducing AGE accumulation and decreasing AGEs-induced oxidative stress could be a useful strategy for treating diabetic nephropathy. In the current study, we established a diabetic mouse model using STZ and tested the protective effects of EU against AGEs-induced renal glucotoxicity.

Numerous studies have reported that most iridoids and phenolic compounds as well as iridoid- and phenolic-rich plant extracts have strong inhibitory activity against AGEs formation [24,25,26]. Moreover, iridoids lowered blood glucose concentrations and improved antioxidant enzyme activity in diabetes [27]. Twenty-four iridoids have been isolated from EU [19] including geniposidic acid, which is present in the bark and has diverse beneficial effects such as anti-atherosclerotic, anti-tumor, and anti-inflammatory [28,29,30] effects. Based on these observations, we also measured the geniposidic acid content of the EU extract using LC/MS, and we plan to perform physiological analyses to verify these findings in the future.

Aminoguanidine and ALT-711 are well-known as an inhibitor and a degrader of AGEs, respectively. However, they induce serious adverse effects and, thus, have poor development prospects [31]. There is a critical need to develop safe and effective drugs to inhibit AGEs formation or breakdown. Recent studies have shown that numerous plant extracts containing bioactive compounds including flavonoids, alkaloids, and tannins can inhibit AGEs formation and cross-linkage [32]. In this study, AGEs-BSA was used to investigate the inhibitory effect of EU on AGEs formation and cross-linkage to collagen, which was measured using an ELISA. EU had a potent inhibitory effect against AGEs-BSA formation, and AGEs-BSA induced cross-links to collagen (Figure 2), suggesting that EU may be a promising intervention agent for diabetic complications.

Numerous research studies have reported that the highly expressed and activated Glo1 protein suppresses AGEs formation induced by high glucose by reducing dicarbonyl accumulation in the kidney [33]. The Glo system is an important pathway for detoxifying reactive dicarbonyl species such as MGO, converting it to D-lactate. Therefore, upregulation of Glo1 expression and activity is an effective strategy for protecting the body against AGEs-induced glucotoxicity. In this study, the expression of Glo1 was significantly downregulated in the STZ-induced diabetic mice, but EU treatment markedly reversed this pattern (Figure 4b). Moreover, EU oral administration significantly increased the Glo1 activity in the diabetic mice (Figure 4e). The IHC analysis also showed strong MGO staining in the diabetic kidney glomeruli; however, EU treatment reduced the deposition of MGO (Figure 5). These results suggest that EU treatment inhibited AGEs formation by increasing Glo1 expression and activity.

Nrf2 is an important transcription factor for regulating antioxidants and detoxification enzymes, such as Glo1 [34]. The strong link between Nrf2 and Glo1 makes Glo1 a potential novel target for controlling AGEs-induced cellular damage mediated by Nrf2 activation. Nrf2 proteins may protect physiological systems to control Glo1. Nowotny et al. [35] reported that increased AGE contents are responsible for the increase in mitochondrial ROS production [35]. ROS generation is well known as a key player in renal cell death. Increased ROS levels enhance the interaction between AGEs and RAGE [36]. The AGEs-RAGE signaling pathway contributes to impairing nitric oxide (NO) activity and causes glomerular sclerosis, leading to tubulointerstitial fibrosis [37].

Moreover, RAGE-AGEs binding activates various intracellular signaling pathways such as mitogen-activated protein kinase (MAPK) signaling, which subsequently activates major transcription factors including nuclear factor (NF)-κB, and induces the production of pro-inflammatory cytokines such as tumor necrosis factor (TNF)-α and interleukin (IL)-6 [38]. Therefore, reducing AGE accumulation and RAGE expression is an effective strategy for treating diabetic complications. In this study, the diabetic mouse kidneys exhibited significantly decreased Nrf2 protein expression and increased RAGE expression, however oral administration of EU significantly reversed this pattern (Figure 4c,d).

The C57BL/6J strain has been reported as a validated model that exhibits a lack of glomerular mesangial expansion in the kidney tissues of the diabetic mice [39]. However, many studies have reported that diabetes induces renal damage in C57BL/6J mice. Shao et al. [40] reported that PAS-positive staining markedly increased in the diabetic C57BL/6J mouse kidney. Renal proximal tubule cells are known to absorb AGEs from the glomerular filtrate and metabolize AGEs [41]. Therefore, the kidney is important for detoxification of AGEs. Moreover, it is well-known that AGEs increase PAS-positive staining in the glomeruli [42]. Furthermore, AGEs were accumulated in the mesangium and glomerular capillary wall of patients with diabetic nephropathy [43]. Based on this observation, the total AGE, histological, and IHC analyses also showed significantly increased glycogen and AGE accumulation in the serum of STZ-induced diabetic mice and the kidney glomeruli tissue, but these changes were prevented by EU treatment (Figure 4f and Figure 5). These results further indicate that EU treatment inhibited AGE accumulation and formation, and reduce RAGE expression by activating the Nrf2 signaling pathway.

Park et al. reported that EU extract has antioxidative effects and regulates blood glucose in type 2 diabetic mice [44]. However, our experiment did not reveal any significant change in blood glucose and HbA1c levels between the different groups of mice. Nevertheless, EU treatment increased the expression of Nrf2, a well-known transcription factor for antioxidant enzymes. Furthermore, in the present study, the diabetes-induced renal glucotoxicity was reversed by inhibiting AGEs formation and RAGE expression. Niu et al. [45] reported that the EU extract showed antidiabetic nephropathy effects without altering blood glucose levels. These differences in results might be attributable to the different extraction methods and animal models used in each experiment.

Park et al. [44] used a water extract of EU and C57BL/Ksj-db/db mice, whereas Niu et al. [45] used a 95% ethanol extract of EU in rats. In our experiments, we used a 70% ethanol extract of EU and C57BL/6J mice. Based on these observations, we cautiously presumed that the blood glucose-lowering effects observed in the study by Park et al. [44] is attributable to some of the hydrophilic components of the EU extract. Furthermore, the renal protection revealed in the study by Niu et al. [45] and our experiments was likely attributable to some hydrophobic components. Further studies should be undertaken to determine differences in the active components of water and ethanol extracts.

Moreover, Park et al. [44] administered the mice a dose equivalent to 1% whole herb/kg of diet and Niu et al. [45] orally treated the rats with 1 g/kg. However, we treated mice with 200 mg/kg EU extract by oral gavage. Among these studies, although ours used the lowest doses of the EU extract, we still observed protective effects against glucotoxicity in the diabetic mouse kidney. These results indicate that both high and low doses of EU improved diabetes-induced glucotoxicity. Although this data is promising, long-term in vivo investigations of the treatment of diabetic nephropathy via regulating glucotoxicity are required as well as the identification of the active compounds.

## 5. Conclusions

EU modulated AGEs and, thereby, reversed AGEs-induced renal damage via Nrf2-Glo pathway activation and amelioration of RAGE expression. Therefore, EU extract could be a potential supplement for the prevention or treatment of diabetes-induced glucotoxicity of the kidney. Further studies are needed to identify the potential chemical metabolites that mediate the anti-glucotoxicity of EU.
